# FGFR4 does not contribute to progression of chronic kidney disease

**DOI:** 10.1038/s41598-019-50669-0

**Published:** 2019-10-01

**Authors:** Ashlee Taylor, Christopher Yanucil, John Musgrove, Melody Shi, Shintaro Ide, Tomokazu Souma, Christian Faul, Myles Wolf, Alexander Grabner

**Affiliations:** 1 Division of Nephrology, Department of Medicine, Duke University School of Medicine, Durham, North Carolina USA; 2 Division of Nephrology, Department of Medicine, The University of Alabama at Birmingham, Birmingham, Alabama USA; 3 Regeneration Next, Duke University, Durham, North Carolina USA; 4 Duke Clinical Research Institute, Duke University, Durham, North Carolina USA

**Keywords:** Chronic kidney disease, Chronic kidney disease, Kidney, Kidney

## Abstract

In chronic kidney disease (CKD), elevated serum levels of the phosphate regulating hormone fibroblast growth factor (FGF) 23 have emerged as powerful risk factors for cardiovascular disease and death. Mechanistically, FGF23 can bind and activate fibroblast growth factor receptor (FGFR) 4 independently of α-klotho, the canonical co-receptor for FGF23 in the kidney, which stimulates left ventricular hypertrophy and hepatic production of inflammatory cytokines. FGF23 has also been shown to independently predict progression of renal disease, however, whether FGF23 and FGFR4 also contribute to CKD remains unknown. Here, we generated a mouse model with dual deletions of FGFR4 and α-klotho, and we induced CKD in mice with either global deletion or constitutive activation of FGFR4. We demonstrate that FGF23 is not capable of inducing phosphaturia via FGFR4 and that FGFR4 does not promote or mitigate renal injury in animal models of CKD. Taken together our results suggest FGFR4 inhibition as a safe alternative strategy to target cardiovascular disease and chronic inflammation in patients with CKD without interrupting the necessary phosphaturic effects of FGF23.

## Introduction

Patients with chronic kidney disease (CKD) develop increased serum levels of the phosphate regulating hormone fibroblast growth factor (FGF) 23^1^. While compensatory elevations of FGF23 maintain normal serum phosphate levels despite reduced renal function, several large epidemiologic studies have demonstrated a powerful and dose-dependent association between elevated FGF23 and increased cardiovascular morbidity, chronic inflammation, greater risk of death^2,3^ and also progression of renal disease^4,5^. FGF23 has been shown to contribute to cardiovascular disease by increasing renal sodium uptake leading to volume expansion and hypertension^6^. As another potential underlying mechanism, we have previously shown that FGF23 can bind and activate FGFR4 independently of α-klotho, the canonical co-receptor of FGF23 in the kidney^7^. Activation of cardiac FGFR4 causes left ventricular hypertrophy^8^, whereas stimulation of FGFR4 in the liver induces hepatic production of inflammatory cytokines^9^ which eventually contributes to the complex reciprocal interplay between chronic inflammation and elevated FGF23 levels in CKD^10^.

CKD is also characterized by α-klotho deficiency and animal models of α-klotho deletion exhibit a distinct CKD-like phenotype consisting of cardiovascular disease, severe alterations in mineral metabolism including elevated serum phosphate and FGF23 levels, vascular calcification and moderate renal injury^11^. Concordantly, α-klotho-hypomorphic (kl/kl) mice are also characterized by accelerated aging and premature death^12^. Since FGF23 has been shown to activate injury-primed renal fibroblasts via FGFR4 and independent of α-klotho^13^, we hypothesized that FGF23-mediated activation of FGFR4 in the kidney directly contributes to the progression of renal injury.

## Results

**Figure 1:**
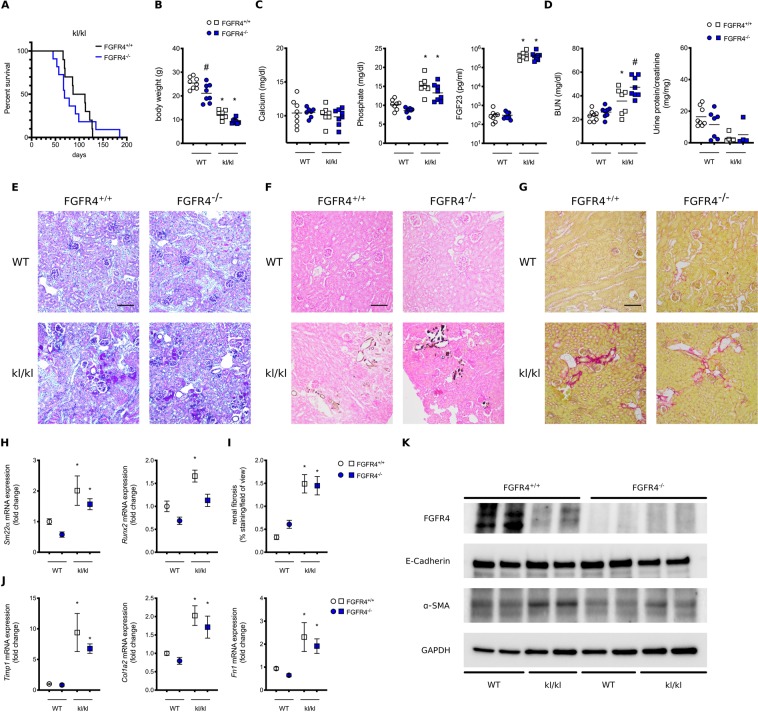
Effect of FGFR4 deletion on kl/kl mice. Compound deletion of FGFR4 does not improve survival of kl/kl mice (10 mice per group) **(A)**. Body weights of wildtype and kl/kl mice with (white) and without FGFR4 (blue) (7–8 mice per group; *p < 0.0001 when compared to wildtype mice) **(B)**. Serum calcium, phosphate and c-terminal FGF23 levels in wildtype and kl/kl mice with (white) and without FGFR4 (blue). (7–8 mice per group; *p < 0.0001 when compared to wildtype mice) **(C)**. Blood Urea Nitrogen (BUN) levels are elevated in kl/kl mice and further increased upon additional FGFR4 deletion in α-klotho hypomorphic animals. No changes in urinary protein-to-creatinine ratios were found (7–8 mice per group; *p < 0.05 when compared to wildtype mice; #p < 0.05 when compared to kl/kl mice)) **(D)**. Representative histopathologic images of kidneys: assessment of renal morphology with Periodic-Acid-Schiff stain **(E)**, Visualization of renal calcification with Von Kossa stain **(F)** and evaluation of renal fibrosis with Picro-sirius Red Stain **(G)**. Renal mRNA expression of markers of calcification including SM22α and Runx2 (7–8 mice per group; *p < 0.05 when compared to wildtype mice) **(H)**. Quantification of renal fibrosis using polarized light microscopy after Picro Sirius Red stain (7–8 mice per group; 8 field of views per kidney; *p < 0.001 when compared to wildtype mice) **(I)**. Renal mRNA expression of markers of fibrogenesis including Timp1, Col1a2 and Fibronectin (7–8 mice per group; *p < 0.05 when compared to wildtype mice) **(J)**. Representative Western Blot images of total kidney lysates showing FGFR4, α smooth muscle actin (α-SMA) and E-Cadherin. GAPDH serves as loading control **(K)**.

First, we analyzed single cell transcriptomics of the mouse^14^ to demonstrate that the kidney expresses all four FGFR isoforms (Supplemental Fig. 1). Second, we aimed to investigate, whether elevated FGF23 levels and klotho independent activation of FGFR4 can contribute to the pathology of kl/kl animals. To test this hypothesis, we generated mice with compound deletion of α-klotho and FGFR4. Here, we report a median life span of 99.5 days in kl/kl mice. Global deletion of FGFR4 in kl/kl mice had no statistically significant effect on survival (median life span 69 days) (Fig. 1A). As illustrated in Fig. 1B, kl/kl mice also suffer from severe growth retardation when compared to wildtype mice, which was also not altered by compound deletion of FGFR4. We did not observe changes in serum calcium; however, serum phosphate and FGF23 levels were significantly elevated in kl/kl mice with no difference between FGFR4 wildtype and FGFR4^−/−^ kl/kl mice (Fig. 1C). Surprisingly, elevated blood urea nitrogen levels in kl/kl mice were further increased by deletion of FGFR4, but urinary protein concentrations were not increased in any group when compared to wildtype mice (Fig. 1D). Histopathological analyses of kidneys revealed mild tubular injury, extensive tissue calcification and fibrosis in kl/kl mice, which remained unchanged upon additional deletion of FGFR4 (Fig. 1E–G). Renal mRNA expression of markers of calcification like Runx2 and SM22α were significantly increased in all kl/kl mice (Fig. 1H). Similarly, renal fibrosis was increased in all kl/kl mice (Fig. 1I) and mRNA expression of markers of fibrogenesis such as Timp1, Col1a2 and Fn1 were elevated in kl/kl kidneys independently of the presence of FGFR4 (Fig. 1J). Western blot analyses of kidney lysates revealed increased expression of α-smooth muscle actin (α-SMA) whereas expression of E-Cadherin was unchanged in kl/kl mice indicating increased fibrosis and myofibroblast formation (Fig. 1K). FGFR4 deletion decreased Runx2 mRNA expression but did not alter any other expression patterns. Interestingly, α-klotho deletion decreased renal FGFR4 expression (Fig. 1K).

**Figure 2:**
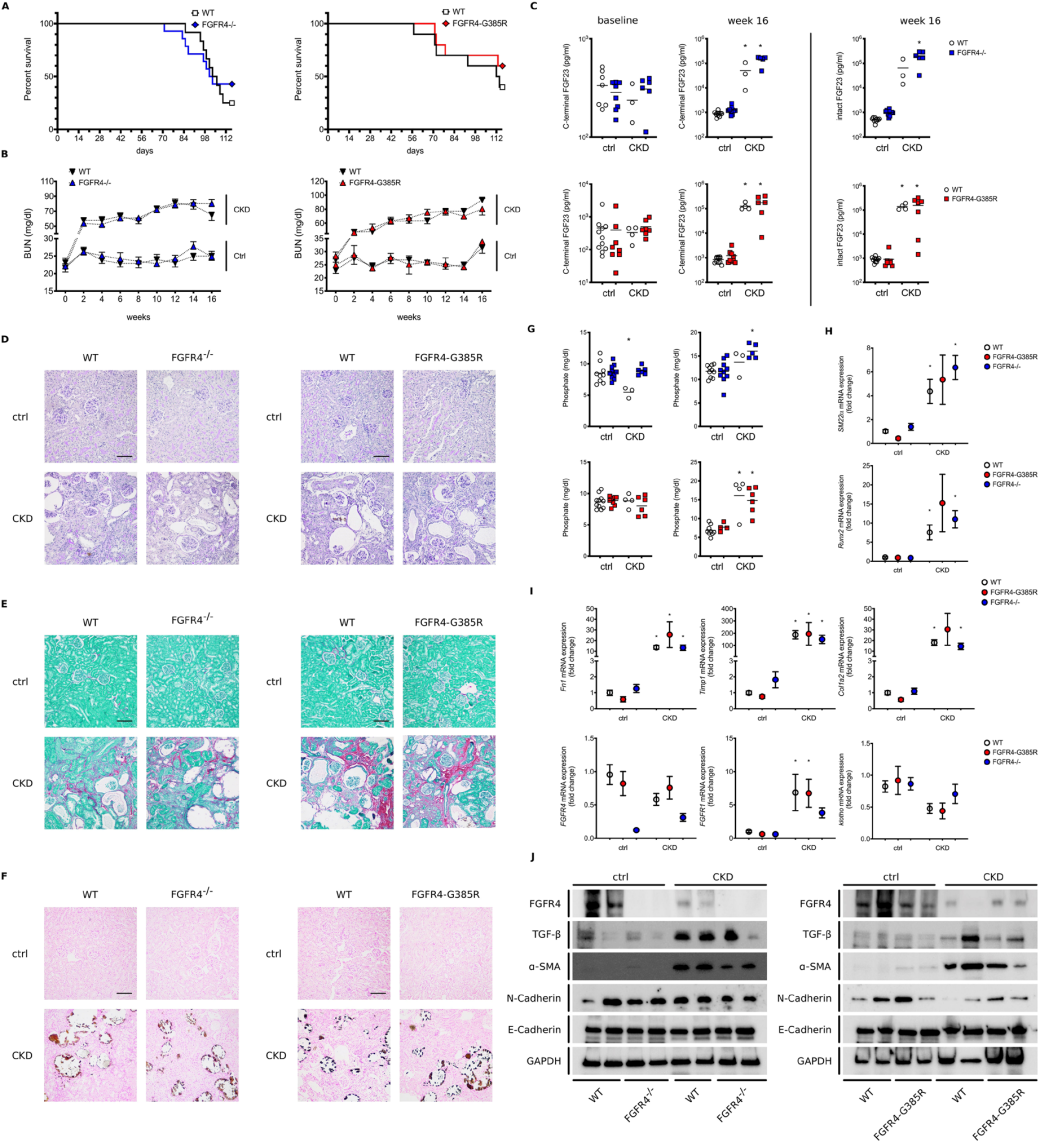
Effect of FGFR4 deletion and FGFR4 activation on the progression of renal injury in CKD. Deletion and gain-of-function of FGFR4 (FGFR4-G385R) have no impact on survival rates in a dietary model of CKD **(A)**. Longitudinal analyses of renal function assessed by serum blood urea nitrogen (BUN) levels. Adenine diet significantly increased serum BUN levels. Statistical analysis using a mixed effect model revealed no differences between wildtype CKD (black arrows) and FGFR4^*−/−*^ CKD (blue arrows) and wildtype CKD (black arrows) and FGFR4-G385R (red arrows) CKD mice respectively **(B)**. Baseline and week 16 measurements of phosphate and C-terminal FGF23 levels in wildtype mice (white circles), FGFR4^*−/−*^ mice (blue squares) and FGFR4-G385R mice (red squares). CKD significantly elevates C-terminal and intact serum FGF23 levels. (n = 3–11 mice per group; *p < 0.001) **(C)**. Representative histopathologic images of kidneys: assessment of renal morphology with Periodic-Acid-Schiff stain **(D)**, Visualization of renal calcification with Von Kossa stain **(E)** and evaluation of renal fibrosis with Picro-Sirius Red/Fast Green Stain **(F)**. CKD induces hyperphosphatemia (n = 3–11 mice per group; *p < 0.05) **(G)** Renal mRNA expression of markers of calcification. CKD kidneys exhibit increased mRNA levels of SM22α and Runx2. (3–11 mice per group; *p < 0.05 when compared to wildtype mice) **(H)**. Renal mRNA expression of markers of fibrogenesis including Timp1, Col1a2 and Fibronectin (3–11 mice per group; *p < 0.05 when compared to wildtype mice). CKD significantly induces fibrosis in wildtype, FGFR4^*−/−*^ and FGFR4-G385R kidneys **(I)**. CKD trends towards a decrease in mRNA expression of α-klotho and FGFR4, but increases FGFR1 mRNA levels (3–11 mice per group; *p < 0.05 when compared to wildtype mice) **(I)**. Representative Western Blot images of total kidney lysates showing FGFR4, TGF-β, α smooth muscle actin (α-SMA), N-Cadherin and E-Cadherin. GAPDH serves as loading control **(J)**.

To further study the potential role of FGFR4 in renal injury we dietary induced CKD in global FGFR4^−/−^ mice and FGFR4-G385R knock in mice (FGFR4-G385R), which carry a gain-of-function mutation of FGFR4. Consistent with the finding in kl/kl mice, neither loss nor constitutive activation of FGFR4 significantly altered survival of CKD mice (Fig. 2A). Similarly, longitudinal assessment of renal function revealed no differences in elevated serum BUN levels of wildtype, FGFR4^−/−^ and FGFR4-G385R mice on adenine diet (Fig. 2B). Correspondingly, serum phosphate (Fig. 2G), intact and C-terminal FGF23 levels were comparably elevated in all CKD groups after 16 weeks. Adenine diet induced profound renal injury as evidenced by glomerular atrophy, tubular dilation and substantial inflammation (Fig. 2D), extensive fibrosis (Fig. 2E) and cortical calcification (Fig. 2F). Concordantly, mRNA expression of markers of fibrosis (Fn1, Timp1, Col1a2) and calcification (Runx2, SM22α) were upregulated in all CKD groups independently of the absence or over-activation of FGFR4 (Fig. 2H,I). CKD kidneys are further characterized by a trend towards decreased mRNA expression of FGFR4 and α-klotho (Fig. 2I), whereas mRNA expression of FGFR1 was significantly increased (Fig. 2I). Western blot analyses confirmed reduced FGFR4 protein expression in CKD (Fig. 2J). Likewise, N-Cadherin expression was decreased in CKD whereas α-SMA and TGF-β were increased in kidney lysates of wildtype, FGFR4^−/−^ and FGFR4-G385R CKD mice (Fig. 2J).

## Discussion

In this brief report, we present several findings that advance our understanding of FGF23, FGFR4 and α-klotho biology. First, we demonstrate that renal FGFR4 expression is highly regulated in CKD and α-klotho deficiency, suggesting that impaired renal function or the uremic milieu consisting of hyperphosphatemia, elevated serum FGF23 levels, α-klotho deficiency or increased oxidative stress directly contribute to the downregulation of FGFR4.

Second, FGFR4 does not significantly modify calcification, renal dysfunction and mortality in kl/kl mice suggesting that hyperphosphatemia and α-klotho deficiency per se are the main drivers of senescence and renal injury. Indeed, compound deletion of the sodium phosphate cotransporter Na-Pi2a or therapeutic interventions targeting phosphate metabolism ameliorate renal dysfunction and significantly improve survival of kl/kl mice^11,15^. Consistently, FGF23 - α-klotho double knockout animals and α-klotho single knock out mice exhibit comparable morbidity and almost identical survival rates^16^. Nevertheless, given the downregulation of FGFR4 in our models, it remains possible that FGF23 mediates its detrimental effects via a different FGFR isoform, dependent or independent of α-klotho^17^.

Third, FGF23 has been shown to mediate its phosphaturic effects mainly via FGFR1 and α-klotho^18^, but also via FGFR3 and FGFR4^19^. Since mice with compound deletion of FGFR4 and α-klotho do not exhibit significantly altered phosphate levels when compared to kl/kl mice, FGF23 is not capable of inducing phosphaturia via FGFR4 independently of α-klotho. Moreover FGFR4^−/−^, FGFR4-G385R and wildtype mice on adenine diet are characterized by similar levels of hyperphosphatemia and comparable elevations of FGF23 suggesting that FGFR4 does not substantially contribute to the regulation of serum phosphate in chronic kidney disease. Consistently, Liu *et al*. have previously shown that FGFR4 deletion does not correct hypophosphatemia in a mouse model of X-linked hypophosphatemic rickets^18^. These results imply FGFR4 targeted therapy to ameliorate cardiovascular disease could be safely used in CKD without obvious adverse effects on mineral metabolism, unlike the effects of anti-FGF23 antibodies^20^.

Recently, FGF23 has been demonstrated to activate injury-primed fibroblasts via FGFR4 thereby inducing TGF-β signaling independently of α-klotho^13^. In their paper, Smith *et al*. used unilateral ureteral obstruction to pre-condition renal fibroblasts, which lead to FGF23-induced pro-fibrotic signaling cascades and myofibroblast activation^21^. However, in our mouse model of dietary induced CKD, we did not observe differences in TGF-β expression, renal fibrosis or markers of fibrinogenesis following FGFR4 deletion or gain of function mutation. This suggests that FGF23-FGFR4 signaling does not play a major role in the development of fibrosis in this particular animal model of CKD.

CKD has been described as a state of α-klotho deficiency and recombinant α-klotho has been shown to mitigate the transition of acute to chronic renal injury whereas α-klotho deficiency exacerbates renal fibrosis and accelerates CKD progression^22,23^. Consistently, adenine diet induced CKD is characterized by reduced renal α-klotho expression and neither deletion nor gain-of-function of FGFR4 restored α-klotho levels in injured kidneys. This suggests that hyperphosphatemia and α-klotho deficiency potentially are drivers of renal injury in the mouse models of CKD that we studied.

In summary, our results suggest that in contrast to CKD-associated pathologies of the liver and the heart, activation of FGFR4 does not contribute to the progression of renal injury in CKD. However, since FGF23 has been shown to correlate with renal injury and to predict the progression of CKD independently of age, proteinuria, renal function and other markers of mineral metabolism^4^, future studies should address the possibility that another FGFR isoform potentially mediates detrimental effects of FGF23 in the kidney.

## Material and Methods

### Generation of FGFR4^−/−^ and kl/kl mice

We generated mice with compound deletion of FGFR4 and α-klotho by mating global FGFR4 knock out (FGFR4^*−/−*^) mice with α-klotho hypomorphic (kl/kl) mice. kl/kl mice were kindly provided by Orson Moe (University of Texas Southwestern Medical Center). We then monitored survival in 10 mice per group. For serologic, molecular and histopathologic analyses, we sacrificed 8 kl/kl mice and 8 mice with compound deletion of FGFR4 and α-klotho at eight weeks of age. 8 Wildtype mice and 8 FGFR4^*−/−*^ mice served as littermate controls.

### Adenine diet mouse model of CKD

We induced renal injury in mice using the adenine diet model of chronic kidney disease. In brief, 8-week old FGFR4^*−/−*^ mice and FGFR4 knock in mice (FGFR4-G385R) were fed a customized chow containing 0.2% adenine, 0.6% calcium and 0.9% phosphate. Wildtype mice on adenine diet and transgenic mice on regular chow served as controls. Heparin plasma was collected every 2 weeks. After 16 weeks, mice were sacrificed, plasma and urine collected and kidneys were prepared for molecular and histopathologic analyses.

### Statistical analysis

Data are presented as mean ± SEM. ANOVA and t tests were used for statistical inference with two-tailed p values < 0.05 considered significant. Sample size was not predetermined by a statistical method, but by extensive laboratory experience from previous publications. We did not use formal randomization for any experiment; for *in vivo* experiments, animals were unbiasedly assigned into different groups. Group allocation was not performed in a blinded manner. One FGFR4-G385R CKD mouse was excluded from data analysis since it did not develop any form of renal injury.

### Study approval

All animal protocols and experimental procedures were approved by the Institutional Animal Care and Use Committees at the University of Miami Miller School of Medicine (FGFR4^*−/−*^ /klotho^*−/−*^ mice) and Duke University (adenine diet in FGFR4^*−/−*^ and FGFR4-G385R mice). All experimental protocols were performed in accordance with relevant guidelines and regulations.

## Supplementary information

Online Supplemental Material
