# Supplementary material for: FGFR4 does not contribute to progression of chronic kidney disease
Source: Sci Rep. 2019 Oct 1;9:14023. doi: 10.1038/s41598-019-50669-0 (PMC6773883; doi:10.1038/s41598-019-50669-0)

**Online Supplemental Material**

**FGFR4 does not contribute to progression of chronic kidney disease**

**Author List:**

Ashlee Taylor^1^, Christopher Yanucil^2^, John Musgrove^1^, Melody Shi^1^, Shintaro Ide^1^, Tomokazu Souma^1,3^, Christian Faul^2^, Myles Wolf^1,4^ and Alexander Grabner^1,3*^

**Affiliations:**

^1^Division of Nephrology, Department of Medicine, Duke University School of Medicine, Durham, North Carolina, USA

^2^Division of Nephrology, Department of Medicine, The University of Alabama at Birmingham, Birmingham, Alabama, USA

^3^Regeneration Next, Duke University, Durham, North Carolina, USA

^4^Duke Clinical Research Institute, Duke University, Durham, North Carolina, USA

***Correspondence:**

Alexander Grabner, MD

Division of Nephrology, Department of Medicine, Duke University School of Medicine; 2 Genome Court, MSRBII, room 1015, Durham, NC, 27713; Tel: +919-684-2873, FAX: +919-684-3011 E-mail: alexander.grabner@duke.edu

**Methods**

*Generation of FGFR4^-/-^ and kl/kl mice*

We generated mice with compound deletion of FGFR4 and **α-**klotho by mating global FGFR4 knock out (FGFR4^-/-^) mice with **α-**klotho hypomorphic (kl/kl) mice. kl/kl mice were kindly provided by Orson Moe (University of Texas Southwestern Medical Center). **α-**klotho hypomorphic mice^1^ and FGFR4^-/-^ kl/kl mice were maintained on a mixed background and received regular chow containing 0.6% calcium and 0.9% phosphate. We then monitored survival in 10 mice per group. For serologic, molecular and histopathologic analyses, we sacrificed 8 kl/kl mice and 8 mice with compound deletion of FGFR4 and **α-**klotho at eight weeks of age. 8 Wildtype mice and 8 FGFR4^-/-^ mice served as controls.

*Adenine diet mouse model of CKD*

Global FGFR4 knock out mice (FGFR4^-/-^)^2^ and global FGFR4 knock in mice (FGFR4-G385R)^3^ were maintained on a C57Bl/6J background. CKD was induced by a customized diet containing 0.2% adenine, 0.6% calcium and 0.9% phosphate (Envigo)^4^. 8-week old mice were fed a control diet for one week and then unbiasedly assigned to either control (9 - 12 mice per group) or adenine diet (11 - 14 mice per group). Every 2 weeks body weights were measured and heparin plasma was sampled via submandibular vein bleed. After 16 weeks, mice were euthanized under isoflurane anesthesia. Heparin plasma was collected via cardiac puncture and tissue was snap frozen or formalin fixed.

*Serology*

Serum BUN and phosphate levels were measured by the Animal Histopathology & Laboratory Medicine Core at the University of North Carolina, which is supported in part by an NCI Center Core Support Grant (5P30CA016086-41) to the UNC Lineberger Comprehensive Cancer Center. Intact and C-terminal FGF23 was assessed using ELISA (Quidel).

*Protein isolation and Western Blot analysis*

For protein extraction from mouse kidneys, tissue was isolated, minced and homogenized in RIPA extraction buffer (50 mM Tris-HCl pH 7.5, 200 mM NaCl, 1% Triton X-100, 0.25% DOC, 1mM EDTA, 1 mM EGTA, protease and phosphatase inhibitors) at 1:10 (w:v). Tissue then was incubated on ice for 30 minutes. Tissue lysates were centrifuged at 21.000g and 4°C for 30 minutes and supernatants were boiled in sample buffer and analyzed by SDS-PAGE and subsequent immunoblotting. A list of primary and secondary antibodies is found in supplemental table 1.

*Supplemental Table 1*

| Antibody | Company | Product number |
| --- | --- | --- |
| GAPDH | Millipore | CB1001 |
| Alpha smooth muscle actin | Cell Signaling | 14968 |
| TGF beta | Cell Signaling | 3711 |
| N-Cadherin | Cell Signaling | 13116 |
| E-Cadherin | Cell Signaling | 3195 |
| FGFR4 | Cell Signaling | 8562 |
| Anti-mouse IgG, HRP-linked Antibody | Cell Signaling | 7076 |
| Anti-rabbit IgG, HRP-linked Antibody | Cell Signaling | 7074 |

*Histology*

For histopathologic analyses, kidneys were formalin fixed overnight, paraffin embedded and serially sectioned (7 µm). Renal sections then were stained with Periodic Acid Schiff. Calcification was assessed using von Kossa stain and renal fibrosis was visualized with Picro Sirius Red and Sirius Red / Fast Green stainings. Fibrosis was quantified across 8 field of views using polarized light microscopy.

*RNA isolation and quantitative real time PCR*

Total RNA was extracted from kidneys with the RNAeasy Mini Kit (Quiagen) and reverse transcribed using the High-Capacity cDNA Reverse Transcription Kit (Applied Biosystems). Quantitative PCR reactions were performed on a QuantStudio 3 real time PCR system (Applied Biosystems) using SsoAdvanced Universal SYBR Green and Probes Supermix (Biorad). Relative gene expression was normalized to expression levels of 18S rRNA and evaluated using the 2^-∆∆Ct^ method. A list of primers and taqman probes can be found in supplemental table 2.

*Supplemental Table 2:*

| gene | Forward primer sequence | Reverse primer sequence |
| --- | --- | --- |
| 18S | TCAAGAACGAAGTCGGAGG | GGACATCTAAGGGCATCAC |
| Fibronectin | AGACCATACCTGCCGAATGTAG | GAGAGCTTCCTGTCCTGTAGAG |
| Timp1 | CTGCTCAGCAAAGAGCTTTC | CTCCAGTTTGCAAGGGATAG |
| Col1a2 | AGCAGGTCCTTGGAAACCTT | AGGGAGTTTCATCTGGCCCT |
| SM22α | TCCAGTCCACAAACGACCAAGC | GAATTGAGCCACCTGTTCCATCTG |
| Runx2 | GCCGGGAATGATGAGAACTA | GGTGAAACTCTTGCCTCGTC |
| FGFR4 (probe) | Thermo Fisher | Mm01341851_g1 |
| **α-k**otho (probe) | Thermo Fisher | Mm00502002_m1 |

*Renal FGFR expression*

Renal FGFR isoform expression has been assessed by analysis of single cell sequencing data using Seurat R package (Supplemental Figure 1). Data is from Park et al^5^.

*Supplemental Figure 1:***
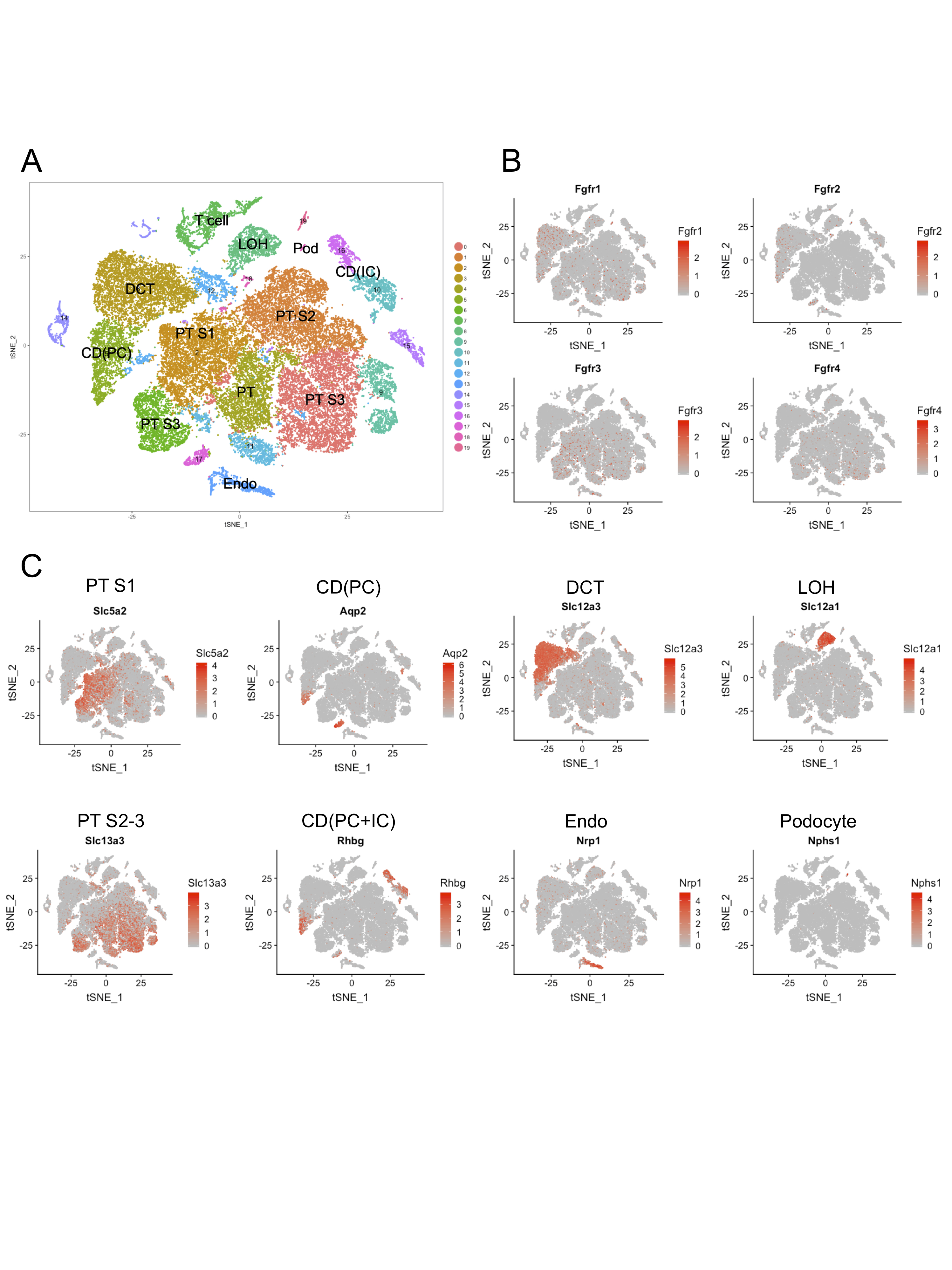
**

Single-cell transcriptomics of the mouse kidneys revealed that all four FGFR isoforms are expressed in the mouse kidney. We analyzed the single-cell transcriptomics data by the group of Susztak (GSE107585; Ref. 1) to identify the expression pattern of FGFRs. (**A** and **B**) Expression pattern of FGFR isoforms in homeostatic kidneys. While gene expressions of FGFR4 and FGFR3 are mainly observed in the proximal tubules, the expression of FGFR2 and FGFR1 genes are more highly expressed in the distal tubular segments (loop of henle to collecting duct). Gene expression levels are visualized as on t-SNE (t-Distributed Stochastic Neighbor Embedding) plot. (**C**) Representative marker gene expression for identification of the cell identity of each cluster. Abbreviation: PT, Proximal tubule segment 1 to 3; DCT, Distal Convoluted Tubule; LOH, Loop of Henle; CD, Collecting duct; PC, Principal Cells; IC, Intercalated Cells; Endo, Endothelium.

**References:**

1. Kuro-o, M. *et al.* Mutation of the mouse klotho gene leads to a syndrome resembling ageing. *Nature* **390,** 45–51 (1997).

2. Weinstein, M., Xu, X., Ohyama, K. & Deng, C. X. FGFR-3 and FGFR-4 function cooperatively to direct alveogenesis in the murine lung. *Development* **125,** 3615–3623 (1998).

3. Seitzer, N., Mayr, T., Streit, S. & Ullrich, A. A Single Nucleotide Change in the Mouse Genome Accelerates Breast Cancer Progression. *Cancer Research* **70,** 802–812 (2010).

4. Jia, T. *et al.* A novel model of adenine-induced tubulointerstitial nephropathy in mice. *BMC Nephrology* **14,** 1–1 (2013).

5. Park, J. *et al.* Single-cell transcriptomics of the mouse kidney reveals potential cellular targets of kidney disease. *Science* **360,** 758–763 (2018).

*Western Blot raw data*

Figure 1:

**
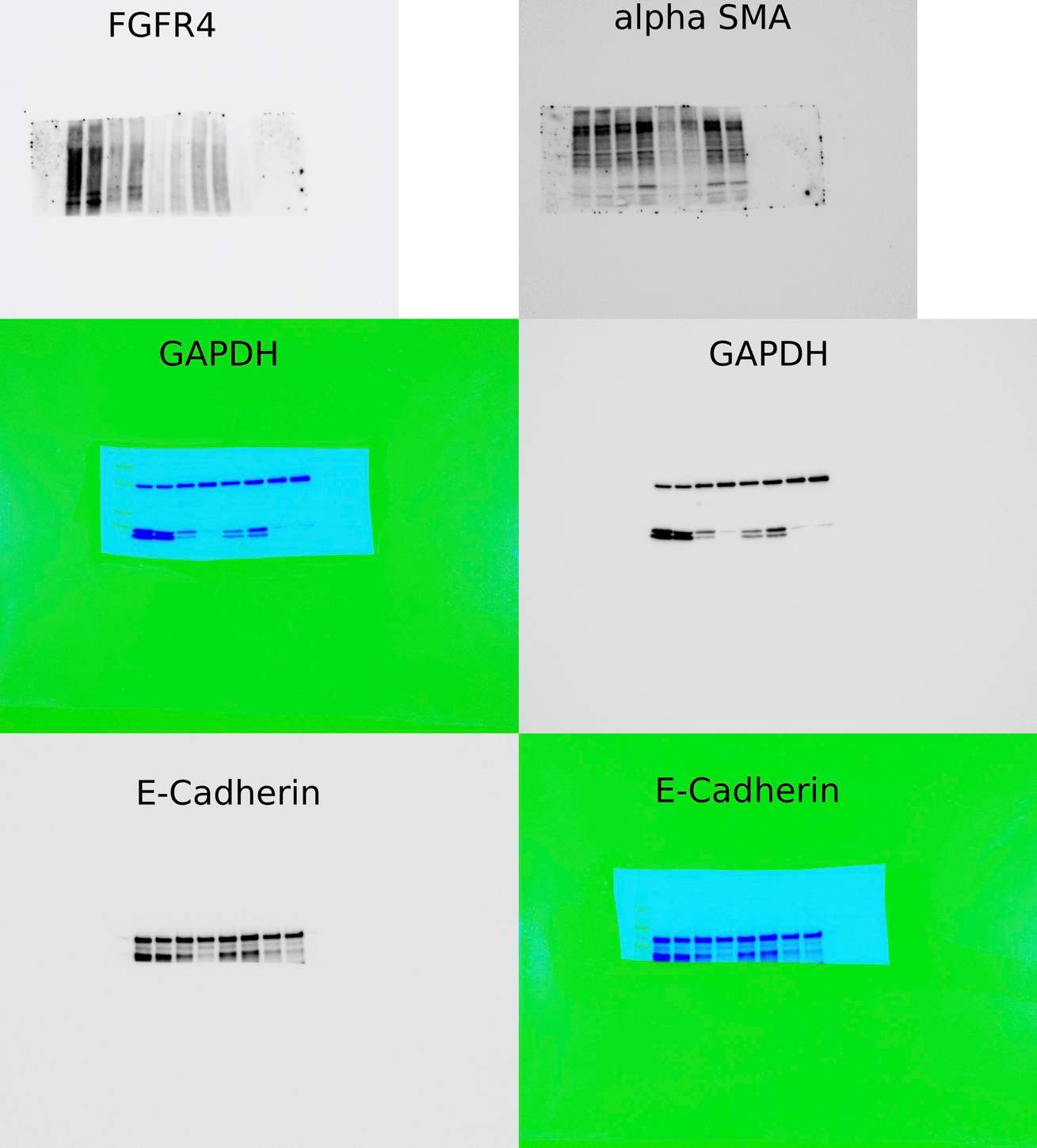
**

Figure 2: (FGFR4^-/-^)

**
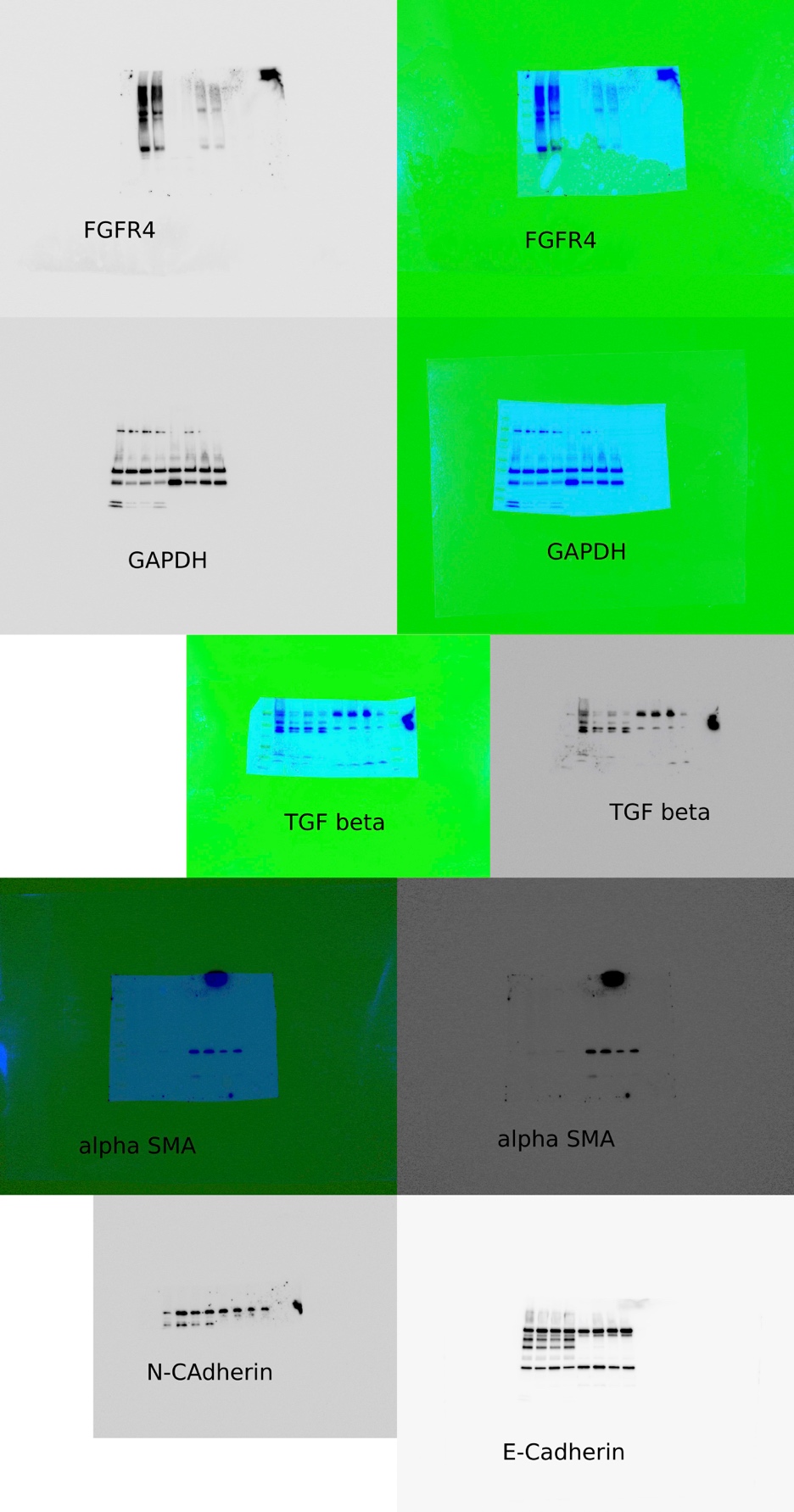
**

Figure 2: (FGFR4-G385R)


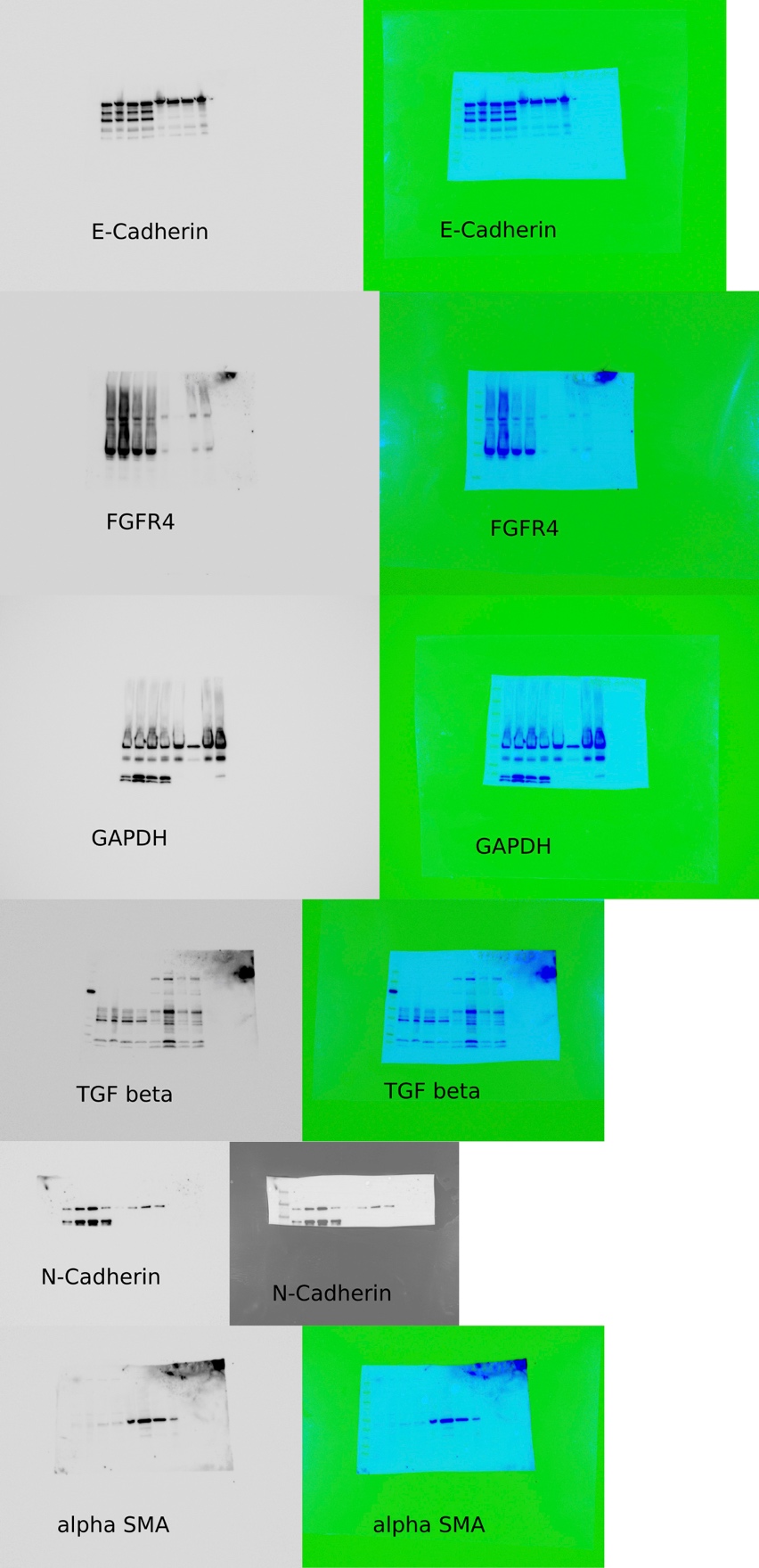

Supplement: Supplementary file 1 — Online Supplemental Material [file 41598_2019_50669_MOESM1_ESM.docx]
